# Impact of seasonality and air pollutants on carotid-femoral pulse wave velocity and wave reflection in hypertensive patients

**DOI:** 10.1371/journal.pone.0172550

**Published:** 2017-02-23

**Authors:** Marina Di Pilla, Rosa Maria Bruno, Francesco Stea, Luciano Massetti, Stefano Taddei, Lorenzo Ghiadoni, Pietro Amedeo Modesti

**Affiliations:** 1 Department of Clinical and Experimental Medicine, University of Pisa, Pisa, Italy; 2 Institute of Clinical Physiology, National Research Council, Pisa, Italy; 3 Institute of Biometeorology, National Research Council, Florence, Italy; 4 Department of Experimental and Clinical Medicine, University of Florence, Florence, Italy; University of Catania, ITALY

## Abstract

**Objective:**

The effects of seasonality on blood pressure (BP) and cardiovascular (CV) events are well established, while the influence of seasonality and other environmental factors on arterial stiffness and wave reflection has never been analyzed. This study evaluated whether seasonality (daily number of hours of light) and acute variations in outdoor temperature and air pollutants may affect carotid-femoral pulse wave velocity (PWV) and pressure augmentation.

**Design and method:**

731 hypertensive patients (30–88 years, 417 treated) were enrolled in a cross-sectional study during a 5-year period. PWV, central BP, Augmentation Index (AIx) and Augmentation Pressure (AP) were measured in a temperature-controlled (22–24°C) room. Data of the local office of the National Climatic Data Observatory were used to estimate meteorological conditions and air pollutants (PM_10_, O_3_, CO, N_2_O) exposure on the same day.

**Results:**

PWV (mean value 8.5±1.8 m/s) was related to age (r = 0.467, p<0.001), body mass index (r = 0.132, p<0.001), central systolic (r = 0.414, p<0.001) and diastolic BP (r = 0.093, p = 0.013), daylight hours (r = -0.176, p<0.001), mean outdoor temperature (r = -0.082, p = 0.027), O_3_ (r = -0.135, p<0.001), CO (r = 0.096, p = 0.012), N_2_O (r = 0.087, p = 0.022). In multiple linear regression analysis, adjusted for confounders, PWV remained independently associated only with daylight hours (β = -0.170; 95% CI: -0.273 to -0.067, p = 0.001). No significant correlation was found between pressure augmentation and daylight hours, mean temperature or air pollutants. The relationship was stronger in untreated patients and women. Furthermore, a positive, independent association between O_3_ levels and PWV emerged in untreated patients (β: 0.018; p = 0.029; CI: 0.002 to 0.034) and in women (β: 0.027; p = 0.004; CI: 0.009 to 0.045).

**Conclusions:**

PWV showed a marked seasonality in hypertensive patients. Environmental O_3_ levels may acutely reduce arterial stiffness in hypertensive women and in untreated patients.

## Introduction

The seasonality of cardiovascular (CV) disease is well known, with acute events occurring more frequently during winter months than in the summertime [1,2,3,4,5,6]. Seasonal variations in blood pressure (BP) [5,7,8,9,10,11] may explain the increase in CV events, such as myocardial ischemia or stroke, during winter. BP and CV disease seasonality may be due to variations in outdoor temperature [5], as well as changes in exposure to sunlight [12], humidity, atmospheric pressure, wind, perception of cold [13], diet changes, water and alcohol intake, physical activity and air pollutants [14]. Conversely, the climate-related variations of arterial stiffness and central BP augmentation, which are key parameters for cardiovascular risk stratification in hypertensive patients and in the general population [15,16,17], have never been investigated. This issues could be of importance since aortic Pulse Wave Velocity (PWV), an indirect measure of large artery stiffness, has a functional component that varies in response to acute stimuli [18] and an increase in Augmentation Index (AIx), and central BP after acute cold exposure has been described [19]. Thus, seasonal variations in PWV and central BP augmentation might play a role in determining the higher rate of CV events during winter.

Another possible factor influencing the increase of BP and CV events during winter is the variation in the levels of air pollutants. Air pollution in urban areas is characterized by both seasonal and daily fluctuations. SO_2_, for example, mainly derived by the heating system of the buildings, shows the highest peak during winter, whilst ozone (O_3_), linked to photochemical smog, reaches the highest concentration during summer and the central hours of the day [20]. The main cause of CV morbidity and mortality due to air pollution is considered to be airborne particular matter with a diameter of less than 2.5 μm (PM_2.5_) [21,22,23], whose increased concentrations have been associated with acute elevations of AIx and PWV after experimental exposure in numerous studies [24,25,26]. However, very few studies have assessed how chronic exposure to outdoor air pollution influences arterial stiffness in humans [27]. To our knowledge, no studies have assessed the acute, real life-effect of air pollutants seasonality on PWV and central BP augmentation.

The aim of the present study is to investigate, in a cross-sectional study, whether variations in seasonality (daily number of hours of light) and environmental variables (mean daily values of outdoor temperature and air pollutants) may acutely affect the gender-specific assessment of carotid-femoral PWV and central BP augmentation.

## Methods

### Study population

For this cross-sectional, retrospective study, data from 731 hypertensive patients aged 30–88 years, 314 untreated (184 men) and 417 treated with antihypertensive drugs (241 men) seeking medical consultation in our outpatient clinic for cardiovascular risk stratification and target organ damage evaluation during a 5-year period (2006–2011) and performing arterial tonometry measurements on the same day, were analyzed.

Inclusion criteria were: age ≥ 18 years; arterial hypertension, defined as blood pressure (BP) ≥ 140/90 mmHg in two previous separate occasions or current antihypertensive treatment; written informed consent[28]. Exclusion criteria were: severe chronic heart failure or hepatic insufficiency; end-stage renal disease; active neoplastic disease; atrial fibrillation. The study conformed to the Declaration of Helsinki and was approved by the local Ethical Committee. All patients gave informed consent for the procedures and the treatment of their clinical data for research purposes.

### Measurements

After enrollment, patients were asked to come to the Hypertension Outpatient Unit after an overnight fasting. A blood sample was drawn for routine examinations (including lipid profile, blood fasting glucose, serum creatinine), according to standard laboratory procedures. Hypercholesterolemia, hypertriglyceridemia and diabetes mellitus were defined according to current guidelines [28] or in the presence of current lipid-lowering or glucose-lowering treatment, respectively. Glomerular filtration rate was estimated using the Modification of Diet in Renal Disease formula (MDRD) [29], and chronic kidney disease (CKD) was defined as glomerular filtration rate <60 mL/min/ 1.73 m^2^. Weight and height were measured and a complete clinical examination was performed; obesity was defined as a body mass index (BMI) >30 kg/m^2^. Current use and dosage of cardiovascular drugs was recorded, as well as medical and pharmacologic history in order to identify cardiovascular risk factors and disease.

Arterial measurements were performed with subjects in supine position in a quiet air-conditioned room (24°C). BP was measured after 10 minutes of rest for three times at 30 seconds intervals by an automatic device (OMRON 950) at the dominant arm. The mean value was calculated from the last 2 measurements [28].

### Pulse wave velocity

A non-invasive measurement studying the structural characteristics of large arteries was performed. Carotid-femoral PWV (cf-PWV) was assessed by arterial tonometry recording waveforms at the femoral and carotid site, sequentially, using a SphygmoCor device (AtCor Medical, Sydney, Australia). A hand-held probe was placed on the selected artery in order to record 10–15 subsequent images. PWV was calculated as the ratio of surface distance between the two recording sites (subtracted distance) to wave transit time calculated by simultaneously recorded electrocardiogram (ECG)[30]. In a subgroup of 320 patients, carotid-radial PWV (cr-PWV) was also measured and aortic-brachial PWV ratio calculated [31].

### Central blood pressure and augmentation index

A hand-held probe was placed on the radial artery from the wrist of the dominant arm and 10 to 15 subsequent images were recorded, as previously described [30]. A validated transfer function was used to transform the radial pressure waveform into the aortic pressure waveform (SphygmoCor, AtCor Medical, Sydney, Australia). Three successive measurements were recorded. Augmented Pressure (AP) was calculated as the difference between the first and the second systolic peak, and the AIx was calculated as the ratio between AP and Pulse Pressure (PP). Because AIx correlates with heart rate, values were also normalized at a heart rate of 75 beats/min.

### Environmental variables assessment

Data on air quality were taken from the European air quality database (airbase v7) [32] maintained by the European Environment Agency that contains air quality monitoring data and information submitted by participating countries. All data on air pollutants, available for the study period at the urban monitoring station PISA Borghetto were considered. Unfortunately there were no available data about PM 2.5 in Pisa since PM 2.5 monitoring started after 2011. Air temperature data in Pisa were retrieved from the Global Summary of the Day dataset provided by the National Climatic Data Centre (NCDC).

### Statistical analysis

Statistical analysis was performed using NCSS8 (NCSS, Kaysville, Utah, USA). The results were expressed as mean ± SD for normally distributed continuous variables and as mean and interquartile range for non-normally distributed variables. Discrete variables were represented as frequency with percentage and counts. Gender differences in the clinical characteristics were analyzed by Student’s test or Wilcoxon Rank Sum test for continuous variables and *X*^*2*^ test for categorical variables. One-way ANOVA was used for the analysis of monthly changes across the year in PWV, AIx and central systolic BP values. Univariate analysis (Pearson coefficient) was performed. In order to analyze factors independently associated with PWV, AIx and central systolic BP, multiple linear regression analysis was performed. Subgroup analysis (men vs women; treated vs non-treated) was also conducted. A P value < 0.05 was considered significant.

## Results

### Clinical characteristics of the study population

Clinical characteristics of the study population are presented in Table 1. Women (n = 306, 41.9%) were older, presented a lower body mass index (BMI) and waist circumference and were more likely smokers. Office brachial systolic and diastolic BP values resulted significantly lower in women than in men. A higher number of men showed a positive family history for cardiovascular events but a lower prevalence of chronic kidney disease (CKD) or impaired fasting glucose (IFG) / diabetes mellitus (DM) than women. 57.2% of the study population was under antihypertensive treatment (241 men) taking on average two BP-lowering drugs (Table 2). As far as BP-lowering drug classes were concerned, a higher use of beta-blockers in women rather than in men was observed.

**Table 1:** Clinical characteristics of the study population.

Variable	Overall population (N = 731)	Men (N = 425)	Women (N = 306)	P value
**Age (years)**	55.7 ± 11.2	54.1 ± 11.3	57.9 ± 10.7	<0.0001
**Height (cm)**	169.3 ± 9.3	174.8 ± 6.8	161.6 ± 6.6	<0.0001
**Weight (kg)**	78.2 ± 14.4	84.6 ± 12.4	69.3 ± 12.2	<0.0001
**BMI (kg/m²)**	26.8 (24.4–29.6)	27.1 (25.3–29.6)	25.8 (23.1–29.6)	<0.0001
**Office systolic BP (mmHg)**	140.9 ± 17.0	142.5 ± 15.6	138.6 ± 18.7	0.002
**Office diastolic BP (mmHg)**	82.1 ± 10.1	83.7 ± 10.2	80.0 ± 9.6	<0.001
**Office PP (mmHg)**	58.7 ± 14.1	58.8 ± 12.9	58.6 ± 15.7	0.84
**Smokers (%)**				0.003
** • Current smokers**	15.3	14.6	16.4	
** • Ex-smokers**	16.6	20.4	11.1	
** • Non smokers**	68.1	65.0	72.5	
**Chronic kidney disease (%)**	9.6	7.7	12.1	0.04
**IFG/DM (%)**	31.1	34.3	26.5	0.03
**Family history for early CV events (%)**	14.9	12.2	18.7	0.02

BMI: Body Mass Index. BP: Blood Pressure. IFG: Impaired fasting glucose. DM: diabetes mellitus. CV: cardiovascular.

**Table 2:** Drug treatment in the study population.

Variable	Overall population (N = 731)	Men (N = 425)	Women (N = 306)	P value
**Antihypertensive treatment (%)**	57.2	57	57.5	0.88
**BP-lowering drugs**	2 (1–2)	2 (1–2)	2 (1–2)	0.80
**Lipid-lowering drugs (%)**	8	6.1	10.5	0.034
**Antidiabetic drugs (%)**	2.6	3.8	1	0.04
**Antithrombotic drugs (%)**	6.7	5.2	8.9	0.05
**Diuretics**	38.7	37.3	40.6	0.51
**ACE-inhibitors**	37.6	41.8	31.6	0.04
**ARBs**	45.5	43.1	49.0	0.25
**Calcium channel blockers**	27.1	32.4	19.4	0.005
**Beta-blockers**	14.7	9.8	21.9	0.001
**Alpha 1 blockers**	4.2	5.8	1.9	0.07
**Aldosterone receptor antagonists**	0.8	0.4	1.3	0.36
**Other drugs**	3.9	6.2	0.6	0.006

BP: Blood Pressure. ACE: Angiotensin Converting Enzyme. ARBs: Angiotensin Receptor Blockers.

Arterial parameters are reported in Table 3. Central PP, but not central systolic BP, resulted significantly higher in women, as well as wave reflection parameters (AP, AIx and AIx75). Cf-PWV values were similar in both sexes, whereas cr-PWV was higher in men than in women.

**Table 3:** Vascular parameters in the study population.

Variable	Overall population (N = 731)	Men (N = 425)	Women (N = 306)	P value
**cf-PWV (m/s)**	8.5 ± 1.8	8.6 ± 1.7	8.5 ± 2.0	0.76
cr-PWV (m/s)*	8.9 ± 1.3	9.1 ± 1.1	8.6 ± 1.4	<0.001
Aortic-brachial PWV ratio*	0.97 ± 0.23	0.95 ± 0.21	1.00 ± 0.25	0.06
**Aortic systolic BP (mmHg)**	130.3 ± 16.5	130.9 ± 15.2	129.5 ± 18.2	0.27
**Mean BP (mmHg)**	103.3 ± 11.9	104.6 ± 11.5	101.6 ± 12.2	<0.001
**Aortic Pulse Pressure (mmHg)**	47.1 ± 13.2	46.0 ± 11.9	48.5 ± 14.6	0.01
**Heart rate (bpm)**	65.2 ± 10.7	64.1 ± 10.6	66.6 ± 10.6	0.003
**Augmentation pressure (mmHg)**	14.2 ± 7.9	12.6 ± 7.5	16.4 ± 8.0	<0.001
**AIx (%)**	28.8 ± 10.7	25.6 ± 10.4	32.1 ± 9.5	<0.001
**AIx75 (%)**	24.1 ± 10.2	20.5 ± 9.4	29.0 ± 9.2	<0.001

cf-PWV: carotid-femoral Pulse Wave Velocity. BP: Blood Pressure. AIx: Augmentation Index.

* available in 320 patients

### Environmental variables

Among the air pollutants studied, O_3_ showed a positive correlation with daylight hours (r = 0.763 p<0.0001), indicating higher levels in summer. The following showed a negative correlation with daylight hours: PM10 (r = -0.304 p<0.0001), CO (r = -0.316 p<0.0001), NO_2_ (r = -0.524 p<0.0001), benzene (r = 0.360 p<0.0001), NO_x_ (r = -0.424 p<0.0001), toluene (r = 0.189 p<0.0001) indicating higher levels in winter. Ethylbenzene and oxylene showed no clear seasonality.

### Pulse wave velocity and central augmentation across the year

Cf-PWV showed an inverse correlation with daylight hours (r = -0.18, p<0.0001). In particular, median monthly cf-PWV value reached the highest values in winter (9.28±2.04 m/s in January and 9.18±2.35m/s in November) and the lowest values in summer (7.99 ±1.47 m/s in June and 8.03±2.02 in July). In the subgroup of patients in which the parameters were available, nor cr-PWV neither aortic-brachial PWV ratio exhibited any clear seasonality (correlation with daylight hours r = -0.09, p = 0.53 and r = -0.08, p = 0.11), though there was a significant increase in its levels(p = 0.006 Kruskal-Wallis One-Way ANOVA on Ranks) in October and November (1.03 ± 0.26 and 1.05 ± 0.26 respectively) in comparison to May and June (0.88 ± 0.15 and 0.90 ± 0.17 respectively).

Brachial and central systolic BP, AP and AIx were not significantly associated with daylight hours. Interestingly, HR showed an inverse correlation with daylight hours (r = -0.11, p = 0.004) and outdoor temperature (r = -0.08, p = 0.04), but not with any of the air pollutants measured. The highest HR values were registered in February (69.3 ± 11.4 bpm) and the lowest in May (63.3 ± 10.8 bpm).

### Determinants of brachial and aortic systolic blood pressure

In the univariate analysis, brachial systolic BP was not correlated with any of the environmental variables, while among clinical variables it showed a slight association with low-density lipoprotein (LDL) cholesterol (r = 0.07, p = 0.06).

Among environmental variables, the univariate analysis demonstrated a positive correlation between central systolic BP and CO (r = 0.10, p = 0.007). Among clinical characteristics, central systolic BP was positively associated to age (r = 0.17, p<0.0001), heart rate (r = -0.12, p = 0.01), and LDL cholesterol (r = 0.08, p = 0.04). A multiple regression analysis, performed including the above-mentioned clinical variables, also adjusted for sex, antihypertensive drugs use, outdoor temperature and daylight hours, showed no significant association between CO and central systolic BP (β: 2.95, p = 0.089; R^2^ full model 0.09).

### Determinants of PWV in the overall population

In the univariate analysis, cf-PWV was directly related to age (r = 0.467, p < 0.001), BMI (r = 0.132, p<0.001), central systolic BP (r = 0.416, p<0.001) and diastolic BP (r = 0.096, p = 0.01). Furthermore, cf-PWV was increased in the presence of hypertriglyceridemia (8.74 ± 1.76 vs 8.43 ± 1.78 m/s, p = 0.04), IFG/DM (8.91 ± 1.90 vs 8.37 ± 1.73 m/s, p<0.001) and CKD (9.68 ± 2.39 vs 8.42 ± 1.72 m/s, p<0.001). Additionally, the following environmental variables were inversely related to cf-PWV: daylight hours (r = -0.176, p<0.001), mean outdoor temperature (r = -0.082, p = 0.03) and O_3_ (r = -0.135, p<0.001). CO (r = 0.096, p = 0.012), N_2_O (r = 0.087, p = 0.022), and NO_x_ (r = 0.076, p = 0.045) showed a positive correlation with PWV.

A multiple linear regression model (Table 4) was created using cf-PWV as dependent variable and including variables significantly related to cf-PWV in the univariate analysis (age, BMI, mean temperature, daylight hours, central systolic and diastolic BP, CO, O_3_) in addition to female sex, BP-lowering drugs, heart rate, hypertriglyceridemia, IFG/DM, CKD, antihypertensive treatment and N_2_O. This analysis revealed that cf-PWV values were independently and directly associated with age, BMI, central systolic BP, heart rate and CKD. Among environmental variables, only the number of daylight hours resulted independently and inversely associated to cf-PWV (β = -0.165, p = 0.002, CI -0.268 to -0.063), accounting for 1.2% of the cf-PWV variance (full model r^2^ = 0.417).

**Table 4:** Multiple regression analysis in the overall population considering PWV as dependent variable.

Variable	β regression coefficient	Lower 95%CI	Upper 95%CI	R^2^	P value
**Female sex**	-0.226	-0.495	0.042	0.003	0.098
**Age**	0.061	0.047	0.075	0.085	<0.001
**BMI**	0.033	0.0008	0.065	0.005	0.045
**Mean T°C**	0.002	-0.327	0.190	0.000	0.859
**Daylight hours**	-0.165	-0.268	-0.063	0.012	0.002
**Aortic systolic BP**	0.040	0.029	0.051	0.060	<0.001
**Diastolic BP**	-0.008	-0.027	0.012	0.001	0.438
**Heart rate**	0.027	0.014	0.039	0.021	<0.001
**IFG/DM**	0.056	-0.221	0.332	0.000	0.693
**Hypertriglyceridemia**	0.145	-0.131	0.422	0.001	0.302
**CKD**	0.747	0.319	1.175	0.014	<0.001
**BP-lowering treatment**	-0.068	-0.327	0.190	0.000	0.603
**NO2**	0.0005	-0.005	0.006	0.000	0.855
**CO**	0.222	-0.284	0.729	0.001	0.389
**Ozone**	0.005	-0.005	0.016	0.001	0.322

CI: confidence interval. BMI: Body Mass Index. T: Temperature. BP: Blood Pressure. IFG: Impaired fasting glucose. DM: diabetes mellitus. CKD: Chronic Kidney Disease.

Among environmental variables, cr-PWV was directly related with CO in the univariate analysis (r = 0.14, p = 0.01). However, the association was no longer significant (regression coefficient 0.073, p = 0.61) in a multiple linear regression model including those variables significantly associated with cr-PWV in the univariate analysis as confounders (age, sex, mean BP, BP-lowering treatment).

Among environmental variables, aortic-brachial PWV ratio was inversely related with O_3_ in the univariate analysis (r = -0.13, p = 0.03). The association lost significance (β -0.001, p = 0.33) in a multiple linear regression model including those variables significantly associated with aortic-brachial PWV ratio in the univariate analysis as confounders (age, sex, BMI, mean BP, total cholesterol, blood fasting glucose, BP-lowering treatment, hypertriglyceridemia, CKD, daylight hours; full model r^2^ = 0.308).

### Determinants of aortic PWV: Subgroup analysis

**Fig 1:**
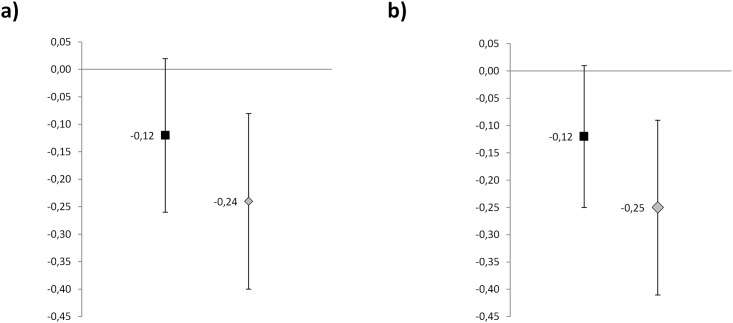
Regression coefficients (95% CL) for daylight hours in multiple regression analysis considering PWV as dependent variable according to BP-lowering treatment (a) and gender (b). Multiple regression models included sex, IFG / DM, hyperTG, CKD, BP-lowering treatment as discrete variables, and age, BMI, Mean T°C, Central SBP, DBP, heart rate, CO, Ozone, NO2 as continuous variables. In panel a), Black squares represent treated patients and gray squares untreated patients. In panel b), Black squares represent men and gray squares represent women.

**Fig 2:**
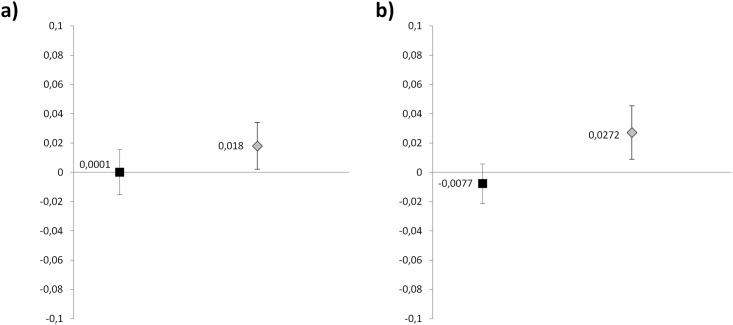
Regression coefficients (95% CL) for daily O_3_ concentrations in multiple regression analysis considering PWV as dependent variable according to BP-lowering treatment (a) and gender (b). Multiple regression models included sex, IFG / DM, hyperTG, CKD, BP-lowering treatment as discrete variables, and age, BMI, Mean T°C, Central SBP, DBP, heart rate, CO, Ozone, NO2 as continuous variables. In panel a), Black squares represent treated patients and gray squares untreated patients. In panel b), Black squares represent men and gray squares represent women.

The same variables included in the multiple linear regression analysis created for the overall population were included in the analysis of the subgroups, with cf-PWV considered as the dependent variable (Figs 1 and 2).

#### BP-lowering treatment

In untreated patients an inverse association between the number of daylight hours and measured values of cf-PWV (β: -0.242; p = 0.003; CI -0.404 to -0.081) was shown, being responsible for 2.7% of the variance of cf-PWV (Fig 1a). Furthermore, high levels of O_3_ were directly associated to cf-PWV values (β: 0.018; p = 0.029; CI: 0.002 to 0.034) (Fig 2a). Age, central systolic BP, heart rate and CKD resulted independent determinants of cf-PWV. Central and brachial BP, AIx and AP exhibited no correlation with daylight hours (r = -0.077, p = 0.179; r = -0.082, p = 0.146; r = 0.043, p = 0.475; r = 0.043, p = 0.475 respectively) and outdoor temperature (r = 0.039, p = 0.492; r = 0.042, p = 0.074; p = 0.222; r = 0.028, p = 0.644 respectively) in both the untreated and treated subgroup, indicating no seasonality. Conversely, in treated patients no significant relationship with daylight hours (β: -0.124, p = 0.072) or any other environmental factor was found (Fig 1a).

#### Gender

In women, multiple linear regression analysis revealed that cf-PWV was independently and inversely associated with daylight hours (β: -0.248; p = 0.003; CI -0.411 to -0.084) (Fig 1b). Daylight hours explained 2.1% of the variance of cf-PWV in women. Furthermore, high levels of O_3_ were directly associated to cf-PWV values (β: 0.027; p = 0.004; CI: 0.009 to 0.045) (Fig 2b). Other independent determinants of cf-PWV were: age, heart rate, central systolic BP, CKD and hypertriglyceridemia. Conversely in men no relationship with daylight hours (β: -0.120 p = 0.068) or any other environmental factor was found (Fig 1b).

### Determinants of central augmentation

The univariate analysis showed no correlation between AIx and environmental factors. Clinical variables correlated with AIx were age (r = 0.306, p <0.0001), BMI (r = -0.171, p<0.0001), brachial SBP (r = 0.116 p = 0.003), central systolic BP (r = 0.364, p< 0.001), total cholesterol (r = 0.141, p<0.001), high-density lipoprotein (HDL) (r = 0.249, p<0.001), creatinine (r = -0.125, p = 0.003), MDRD (r = -0.093 p = 0.027). Furthermore, AP (mean value 14.2±7.9 mmHg) was related to age (r = 0.45, p<0.001), BMI (r = -0.13, p = 0.001), central SBP (r = 0.68, p<0.001), heart rate (r = -0.38, p<0.001), total cholesterol (r = 0.15, p<0.001), LDL (r = 0.09, p = 0.03), HDL (r = 0.19, p<0.001), estimated glomerular filtration rate (r = -0.18, p<0.001).

No significant correlation was found between AIx, AP and daylight hours, mean temperature and air pollutants, neither in the univariate analysis, nor in the multiple regression analysis adjusted for confounders.

## Discussion

This cross-sectional analysis aimed at detecting variations in seasonality (expressed as daily number of hours of light) and environmental variables (represented by mean daily values of outdoor temperature and air pollutants) in a population of hypertensive patients and how they may affect carotid-femoral PWV and central augmentation. The main result of the study is that the daily number of hours of light independently affect measurements of cf-PWV in hypertensive patients. The association was stronger in women and in untreated patients. Among air pollutants, a positive and significant association between outdoor O_3_ concentrations and cf-PWV emerged in untreated hypertensive patients, an association occurring regardless of the shown seasonal variations of both parameters. Furthermore, in women a significant and positive association between aortic stiffness and outdoor O_3_ concentrations was found, suggesting that this subgroup might be more vulnerable to the effects of acute exposure to air pollution.

The seasonality of CV disease has been attributed to variations in BP, secondary to changes in outdoor temperature and other environmental variables [14]. Our data support the hypothesis of a role of seasonal variations in cf-PWV in determining the higher rate of CV events during winter. While climate-related variations of arterial stiffness have never been investigated, in healthy young volunteers full-body cold exposure for 30 minutes at 4°C increased brachial and central systolic BP and wave reflection (expressed as AIx), possibly via sympathetic-induced vasoconstriction, in agreement with our results [19]. Cold-induced sympathetic activation might be a possible mechanism explaining seasonality of cf-PWV. Indeed, resting cf-PWV and muscle sympathetic nerve activity are positively related in humans [33] and maneuvers inducing sympathetic activation, such as mental stress, also induce endothelial dysfunction, which in turn might acutely increase arterial stiffness [18]. However, this mechanism does not appear to play a relevant role, since in our study the correlation between outdoor temperature and cf-PWV was no longer significant in the multiple regression model. Interestingly, both cf-PWV and daylight hours are associated to heart rate, a surrogate marker for sympathetic nervous system activity, despite the association between arterial stiffness and seasonality remain significant when adjusted for heart rate in the multivariate analysis. Furthermore, the association between cf-PWV and daylight hours resulted significant irrespective of brachial and central systolic BP, suggesting a selective effect on the vasculature that is independent of systemic hemodynamics. However, an increase in cf-PWV may precede and be the causative mechanism for BP elevation [34]and then for the seasonality of BP. Other plausible explanations might be related to the lifestyle modifications (dietary changes, physical activity, sleep pattern), which may occur over the year. Twenty-four hour urinary sodium excretion is increasingly higher as seasons become colder, either in mice or in humans [35]. Interestingly, sodium intake is associated to arterial stiffness independently of changes in mean arterial pressure [36], thus providing a possible explanation for our findings. Furthermore, a higher number of daily steps in summer rather than in winter was demonstrated [37], possibly leading to reduced arterial stiffness. Finally, a longer exposure to sunlight during warmer months results in an elevated biosynthesis of the active form of vitamin D, 1,25(OH)_2_D_3,_ possibly leading to a reduction in arterial stiffness [35]. However contrasting data exist on the role of Vitamin D for cardiovascular prevention, BP reduction and arterial destiffening [38,39,40].

Another interesting finding of our study is that cf-PWV was inversely associated to O_3_ levels, which was especially evident in untreated hypertensive patients and in women. A number of studies [24,25,26] have shown an increase both in AIx and cf-PWV after exposure to PM2.5, considered as the main cause of CV morbidity and mortality due to air pollution [21,22,23]; unfortunately, PM2.5 levels were not available for the present study. However, other substances may have a detrimental effect on vascular function and structure as well. In a cohort of young adults, long-term exposure to NO_2_ and SO_2_ was associated to a significant increase in cf-PWV and AIx [27]. Indeed, oxidative stress-induced endothelial dysfunction is, together with autonomic nervous system imbalance, hypothalamus-pituitary—adrenal axis, release of pro-inflammatory mediators and of prothrombotic pathways, one of the main mechanisms linking air pollution with cardiovascular disease [41]. Since endothelial dysfunction contributes to the functional component of large artery stiffness [42], it is likely that the observed effect of air pollutants on vascular stiffness might be mediated by increased oxidative stress. Chronic exposure to high O_3_ levels may induce endothelial dysfunction in in rat coronary arteries [43], whereas contrasting data have been found after experimental exposure in healthy volunteers[26,44]. To our knowledge, the acute effect of environmental O_3_ on large artery stiffness has been shown for the first time by this analysis. Antihypertensive therapy might have masked this effect, since most antihypertensive drugs affect arterial stiffness, directly or indirectly [45]. Furthermore, this study suggests that women may be more vulnerable than men to the effect of air pollution, in parallel to what demonstrated for noise pollution [46]. On the contrary, the majority of data demonstrating the relationship between air pollution and cardiovascular diseases shows positive correlations, more evident in males [47]. These contrasting results may be related to higher exposure level of air pollution and tobacco smoking in men than women in Asian Countries, where the majority of the studies regarding this topic are conducted.

## Strengths and limitations

To our knowledge, this is the first study investigating the impact of seasonality and air pollutants on carotid-femoral PWV in a population of hypertensive patients, both treated and untreated with BP-lowering drugs. An adequate sample size is another strength of the study.

The main study limitation is the cross-sectional design of the study. Prospective studies are required in order to firmly demonstrate the role of cf-PWV in determining the higher rate of cardiovascular events during winter. Furthermore, the study did not include objective measurements of 24h-BP, which might have revealed a relationship between arterial stiffness and circadian BP pattern, which is peculiarly influenced by seasonality [10,11]. Unfortunately, for this study it was not possible to measure a number of air polluntants, including PM 2.5, as already discussed, and air metals: penetration to the systemic circulation of diffusible components such as soluble metals, such as nickel, may also lead to potentiation of inflammatory responses and to impaired vascular function [41]. Furthermore, markers of oxidative stress were not available in the study population. Finally, our study did not include the objective evaluation of a number of possible determinants of seasonal variations of cf-PWV, including sodium intake, physical activity, sleep duration and vitamin D levels.

## Conclusions

Seasonality affects carotid-femoral PWV in hypertensive patients. Arterial stiffness, evaluated as cf-PWV, is considered as a form of subclinical hypertensive organ damage according to current guidelines for hypertension management [28] and it might improve risk stratification in individuals at intermediate cardiovascular risk [16]. Thus, since many authors claim cf-PWV use in daily clinical practice, our findings suggest that seasonality should be taken into account when assessing for arterial stiffness for CV risk stratification or in clinical trials. Furthermore, cf-PWV seasonal increase might play a role in determining the higher rate of CV events during winter. Finally, future prospective and mechanistic studies are necessary to confirm the possible detrimental effect of outdoor O_3_ concentrations on cf-PWV suggested by our analysis, and elucidate mechanisms of its gender-specific effect.
